# The relationship of dysfunctional breathing with the ideas about the COVID-19 pandemic among the healthy population of Russia

**DOI:** 10.1192/j.eurpsy.2022.1255

**Published:** 2022-09-01

**Authors:** J. Koniukhovskaia, E. Pervichko, O. Mitina, O. Stepanova, V. Petrenko, I. Shishkova, E. Dorokhov

**Affiliations:** 1 Pirogov Russian National Research Medical University, Clinical Psychology Department, Moscow, Russian Federation; 2 Lomonosov Moscow State University, Psychology, Moscow, Russian Federation; 3 Ryazan State Medical University named after I.P. Pavlov, Faculty Of Clinical Psychology, Ryazan, Russian Federation

**Keywords:** Naimigen questionnaire, Covid-19 pandemic, dysfunctional breathing

## Abstract

**Introduction:**

Dysfunctional breathing is experienced as a feeling of “difficulty in inhaling” and shortness of breat , which may be similar to the symptoms of coronavirus infection (Gavriatopoulou et al., 2020). The conditions of the COVID-19 pandemic create an increased level of anxiety and attention to respiratory sensations, which becomes a favorable ground for the occurrence of dysfunctional breathing.

**Objectives:**

To examine the relationship of ideas about the pandemic with the occurrence of dysfunctional breathing in the Russian population during the COVID-19 pandemic.

**Methods:**

The Naimigen Questionnaire (Van Dixhoorn, Duivenvoordent, 1985) and the author’s socio-demographic questionnaire were used, which included questions about personal experience of the pandemic. The study was conducted online from April 27 to December 28, 2020. It was attended by 1,362 people from all regions of Russia, including 1,153 women and 209 men aged 15 to 88 years (38.3±11.4).

**Results:**

It was found that respondents who are more confident in the danger of coronavirus have more respiratory difficulties (N=517;NQ=19±10.6) compared to those who consider its danger exaggerated (N=454,NQ=15.9±9.2,p=0.000). Respondents who are completely convinced of the absence of a condemnation for COVID-19 disease have less pronounced dysfunctional breathing (N=331,NQ=15.26±9.5), compared to those who sure about it (N=88,NQ=19.16±10.05, p=0.007). Respondents who have relatives ill COVID-19 (N=430) have a higher score on NQ (18.6±10.5), compared with those dont have (N=932, NQ=17.1±9.7, p =0.011).

**Conclusions:**

The dysfunctional breathing is associated with the respondents’ beliefs about the danger of coronavirus and the expectation of stigmatization in COVID-19 disease, as well as with the experience of COVID-19 disease among relatives.

**Disclosure:**

Research is supported by the Russian Science Foundation, project No. 21-18-00624.

